# mt-tRNAs in the polymerase gamma mutant heart

**DOI:** 10.20517/jca.2023.26

**Published:** 2023-10-18

**Authors:** M. Bilal Bayazit, Ashley Francois, Erin McGrail, Federica Accornero, Matthew S. Stratton

**Affiliations:** 1 Department of Physiology & Cell Biology, Davis Heart and Lung Research Institute, The Ohio State University, Columbus, OH 43210, USA; 2 Center for RNA Biology, The Ohio State University, Columbus, OH 43210, USA

**Keywords:** Cardiac aging, mt-tRNA, UPR^MT^, POLG

## Abstract

**Introduction:**

Mice harboring a D257A mutation in the proofreading domain of the mitochondrial DNA polymerase, Polymerase Gamma (POLG), experience severe metabolic dysfunction and display hallmarks of accelerated aging. We previously reported a mitochondrial unfolded protein response (UPT^mt^) - like (UPR^mt^-like) gene and protein expression pattern in the right ventricular tissue of POLG mutant mice.

**Aim:**

We sought to determine if POLG mutation altered the expression of genes encoded by the mitochondria in a way that might also reduce proteotoxic stress.

**Methods and Results:**

The expression of genes encoded by the mitochondrial DNA was interrogated via RNA-seq and northern blot analysis. A striking, location-dependent effect was seen in the expression of mitochondrial-encoded tRNAs in the POLG mutant as assayed by RNA-seq. These expression changes were negatively correlated with the tRNA partner amino acid’s amyloidogenic potential. Direct measurement by northern blot was conducted on candidate mt-tRNAs identified from the RNA-seq. This analysis confirmed reduced expression of MT-TY in the POLG mutant but failed to show increased expression of MT-TP, which was dramatically increased in the RNA-seq data.

**Conclusion:**

We conclude that reduced expression of amyloid-associated mt-tRNAs is another indication of adaptive response to severe mitochondrial dysfunction in the POLG mutant. Incongruence between RNA-seq and northern blot measurement of MT-TP expression points towards the existence of mt-tRNA post-transcriptional modification regulation in the POLG mutant that alters either polyA capture or cDNA synthesis in RNA-seq library generation. Together, these data suggest that 1) evolution has distributed mt-tRNAs across the circular mitochondrial genome to allow chromosomal location-dependent mt-tRNA regulation (either by expression or PTM) and 2) this regulation is cognizant of the tRNA partner amino acid′s amyloidogenic properties.

## Graphical Abstract

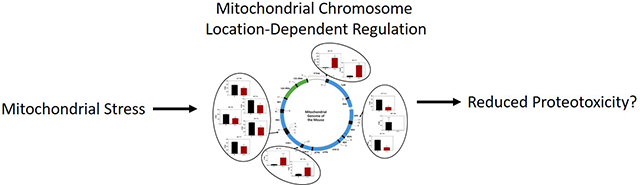

## INTRODUCTION

Mitochondrial function and mitochondria-derived signals play central roles in many pathologies, including those associated with aging. Several mitochondrial diseases in humans are attributed to mutations in the mitochondrial DNA polymerase, Polymerase Gamma (POLG) (reviewed in^[1]^). Mouse models recapitulating these POLG mutations display severe metabolic dysfunction and accelerated aging phenotypes including hair loss, weight loss, brittle bones, cardiac dysfunction, and early mortality^[2–9]^.

We recently reported evidence for a pattern of protein and nuclear-encoded gene expression similar to the mitochondrial unfolded protein response (UPR^mt^) in the POLG D257A mutant mouse^[10]^. POLG lacks proofreading capacity in this mutant, resulting in reduced mitochondrial DNA integrity^[11]^ and severe dysfunction. In mammals, building on well-characterized pathways in lower-level organisms (e.g., *C. elegans*), UPR^mt^ is thought to be activated when mitochondrial dysfunction impedes the ability of the mitochondria to import AFT4/ATF5/CHOP which then become available to enter the nucleus and function as transcription factors. A host of nuclear-encoded genes are then activated that sum to reduce mitochondrial dysfunction by reducing proteotoxic stress (increase expression of mitochondrial chaperones and proteases), reducing reactive oxygen species, increasing mitochondrial protein import, and activating innate immunity (reviewed in^[12,13]^). Our previous analysis of the POLG mutant indicated post-transcriptional activation of proteins associated with mitochondrial protein expression and complex assembly along with decreased expression (mRNA and protein) of metabolic complex subunits^[10]^. Thus, the UPR^mt^-like response in POLG does not completely recapitulate UPR^mt^ but is a pattern consistent with reducing proteotoxic stress similar to UPR^mt^. For instance, POLG mutants show increased protein expression of mitochondrial proteases (AFG3L2, PMPCB), mitochondrial ribosomal proteins (MRPS30, MRPL21, MRPL37, MRPS14, DAP3), translation regulators (ATAD3A, EEFA1A, GUF1), mitochondrial chaperones (PHB1, PHB2, PET100, STOML2), enzymes required for protein folding (PPIB, TMX1), mitochondrial protein importers (TOMM40, MTX1), and factors necessary for complex assembly (ECSIT, DNAJC11, COQ4, COA3, COA5)^[14]^. Given the presence of rRNAs and tRNAs in the mitochondrial genome, we sought to determine if POLG mutation altered the expression of genes encoded by the mitochondria in a way that might also reduce proteotoxic stress.

## MATERIALS AND METHODS

### Animals

As reported in^[10]^, PolG D257A mutant mice were acquired from the Jackson Laboratory (B6.129S7(Cg)-Polgtm1Prol/J, deposited by Dr. Tomas A Prolla) and housed in the Davis Heart and Lung Research Institute vivarium. Wild type (WT) and homozygous POLG D257A mutants (POLG) were generated by heterozygous breeding. Animals were maintained on a 12:12 light:dark schedule and had free access to water and food (Teklad 7012). All animal care and experimental procedures were in accordance with the Ohio State University IACUC and NIH ethical use of animal guidelines. All samples included in this analysis were from female mice at 9-10 months of age.

### RNA sequencing and analysis

RNA sequencing and analysis through differential expression were performed as reported in^[10]^. Briefly, Poly(A) RNA sequencing library was prepared from DNAse treated RNA from flash frozen RV tissue [female WT (4) and POLG (4) at 9-10 months of age] following Illumina’s TruSeq-stranded-mRNA sample preparation protocol. Paired-ended sequencing was performed on Illumina’s NovaSeq 6000 sequencing system. Reads were mapped to MM10 with HISAT2^[15]^ assembled and normalized in StringTie^[16]^. Global differential expression analysis was conducted in EdgeR^[17]^. Normalized gene expression values (generated with Stringtie) for genes encoded by the mitochondrial DNA were recovered from transcriptome-wide RNA-seq data. A *t*-test was used to compare the expression between control and POLG mutant RVs. We also considered the edgeR calculated p value from the global RNA-seq analysis and is reported together with the *t*-test value in Supplementary Table 1. These tests weigh the magnitude of change and within-group variability differently (edgeR does not consider within-group variability). Genes showing expression changes of *P* < 0.05 by *t*-test and *P* < 0.05 by edgeR or *P* < 0.1 by *t*-test and *P* < 0.0001 by edgeR were determined to be significantly changed. Library generation, sequencing, and initial differential expression analysis was conducted by LC Sciences (Houston, TX).

### mt-tRNA northern blot

Cardiac tissue was homogenized in TRIzol using a tissue homogenizer (Next Advance). Total RNA was then isolated using the TRIzol-chloroform method. RNA was precipitated with isopropanol and washed with ethanol. A total of 2 μg of RNA was resuspended in RNA urea loading dye, dissolved in 8M urea 8% polyacrylamide gels (80 V, 150 min), and electroblotted (80 V at 4 °C, 90 min) into Zeta-probe nylon membranes (Bio-Rad). The membrane was then UV-cross-linked for 1 min. DNA oligonucleotides in reverse complementarity to the tRNA were radioactively labeled with 32P γ-ATP. Northern blot hybridization was performed according to manufacturer specifications (Bio-Rad) using the 32P-labeled oligonucleotides. After hybridization, membranes were exposed overnight to a phosphorimager screen. Blots were analyzed using a Typhoon FLA 9000 scanner and the ImageQuant TL software (GE Healthcare). The following probes were used for Northern hybridization^[18]^:

MT-TP TCAAGAAGAAGGAGCTACTCCCCACCACCA,

MT-TY TGGTAAAAAGAGGATTTAAACCTCTGTGTT,

MT-TL1 TATTAGGGAGAGGATTTGAACCTCTGGGAA.

### Statistics

Statistical analysis was conducted in GraphPad Prism V7.05 using linear regression and *t*-test functions. All *t*-tests were unpaired and two-tailed.

## RESULTS

### POLG mutant-induced gene expression changes in mitochondria-encoded genes

**Figure 1.**
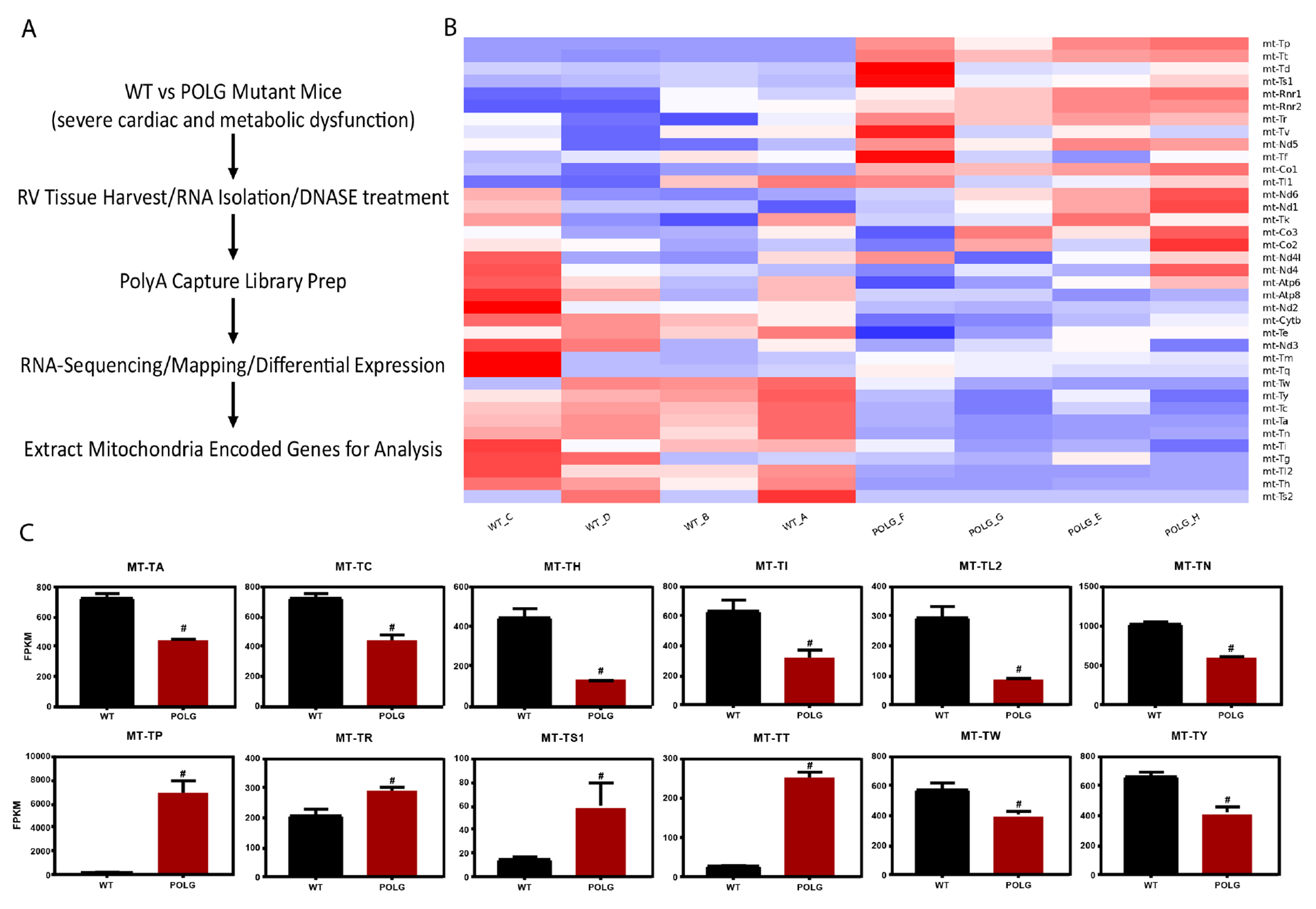
Polymerase gamma mutation drives altered mitochondrial gene expression in the cardiac right ventricle. Experimental workflow is depicted (A). Row normalized expression of the 37 mitochondria encoded genes is shown in heatmap format (B). Twelve of the 22 mt-tRNAs showed altered expression in the POL mutant (C).

Expression values for mitochondria-encoded genes were recovered from whole genome RNA-seq data generated from WT and POLG mutant mouse right ventricular cardiac tissue [Figure 1A] and are displayed in heatmap format [Figure 1B]. Seventeen of the 37 mitochondrial genes showed significant expression changes in the POLG mutant compared to WT control mice [Supplementary Table 1]. Importantly, gene expression changes were not unidirectional. Among non-tRNA genes, we observed robustly increased expression of rRNAs (mt-Rnr1 and mt-Rnr2) and increased expression of cytochrome C oxidase subunit I (mt-Co1) and NADH dehydrogenase subunit 5 (mt-Nd5). Cytochrome b (mt-Cytb) showed a modest but significant reduction in expression in the POLG mutant.

**Figure 2.**
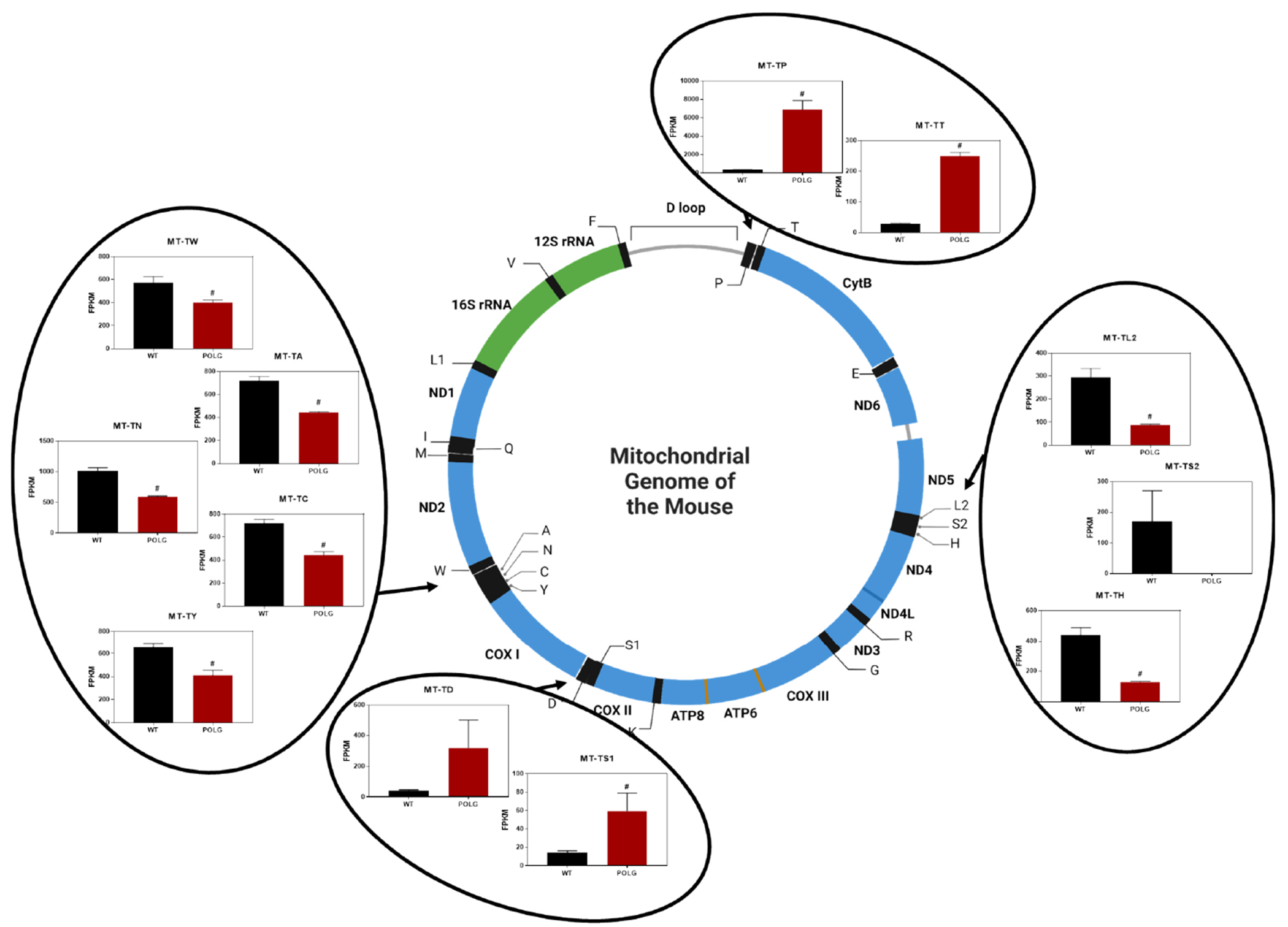
Chromosomal location-specific effect of POLG mutation on mt-tRNA expression as measured by RNA-Seq. Expression of clustered mt-tRNAs in WT and POLG mutant mice is presented in relation to location of encoding within the mitochondrial genome. A pattern of 4 local clusters of mt-tRNAs emerges where each mt-tRNA in the cluster shows similar changes in gene expression in response to POLG mutation (center depiction of mitochondrial chromosome derived in BioRender).

Remarkably, 12 of 22 mt-tRNAs were significantly altered in the POLG mutant [Figure 1C]. When the expression of these mt-tRNAs in POLG mutant *vs*. WT mice is displayed along the mitochondrial genome (circular), a striking location-dependent pattern emerges [Figure 2]. Specifically, 4 clusters of mt-tRNAs were identified for which the expression of the mt-tRNAs in the cluster moved in the same direction during mitochondrial stress. Cluster 1 holds MT-TP (encoding tRNA proline) and MT-TT (tRNA threonine), both of which are significantly activated in the mutant. Cluster 2 holds MT-TL2 (tRNA leucine 2), MT-TH (tRNA histidine), and MT-TS2 (tRNA serine 2), which all show decreased expression (MT-S2 did not meet significance criteria). Cluster 3 holds MT-TD (tRNA aspartic acid) and MT-TS1 (tRNA serine 2) which increased expression (MT-TD did not meet significance criteria). Cluster 4 holds MT-TW (tRNA tryptophan), MT-TA (tRNA alanine), MT-TY (tRNA tyrosine), MT-TN (tRNA asparagine), and MT-TC (tRNA cysteine), all of which showed decreased expression.

**Figure 3.**
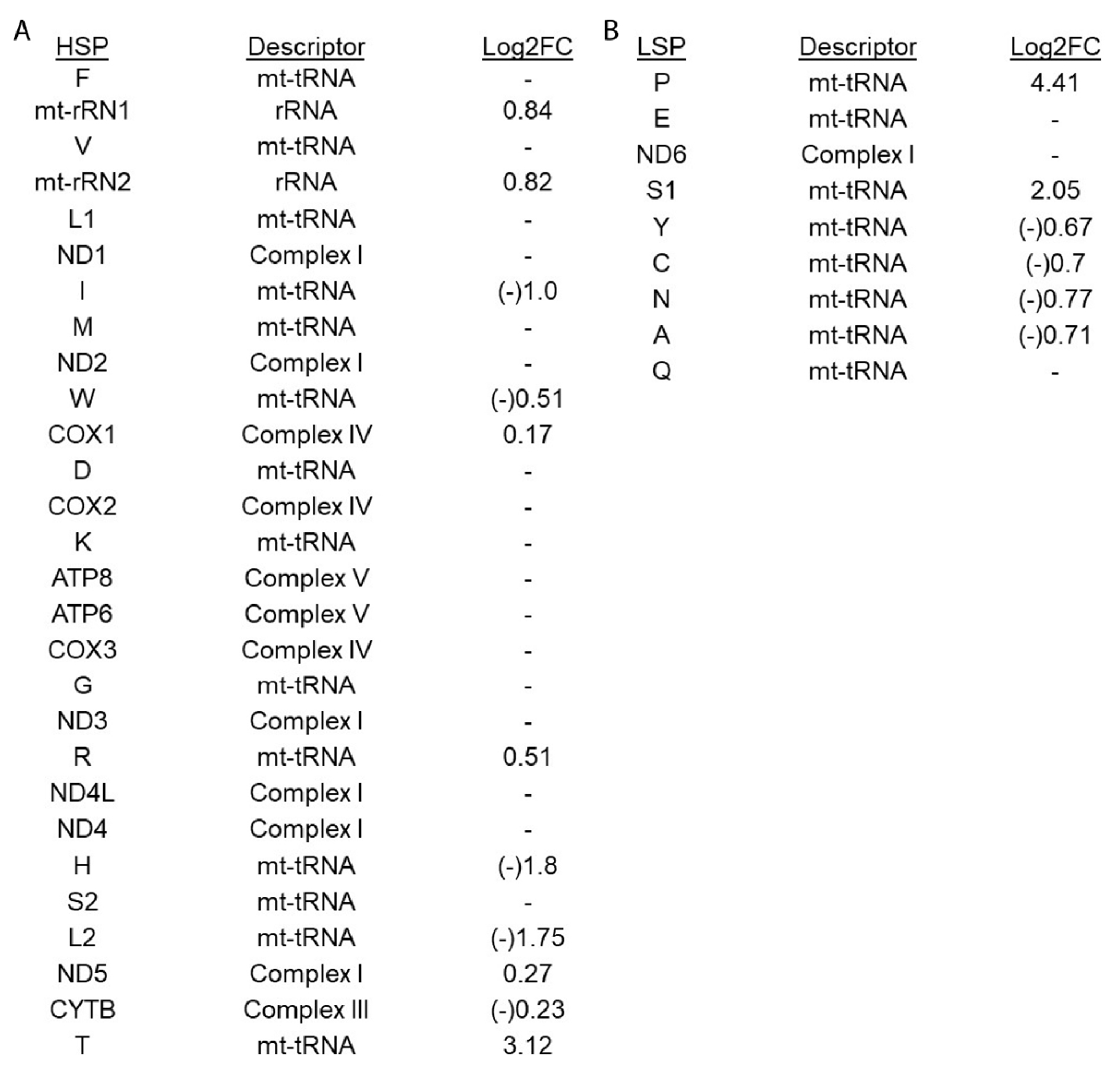
POLG-dependent changes in mitochondrial gene expression are not associated with promoter usage. Genes are depicted based on expression from mitochondrial promoters HSP (A) and LSP (B). Descriptor column provides information related to gene function. Log2FC in POLG mutant relative to control RV tissue show opposite direction of gene expression regulation from the same promoterr.

Mitochondrial genes are expressed from only two transcription start sites (the existence of a third, alternate HSP is controversial) and specific genes are spliced from the larger transcript containing up to 25 genes (reviewed in^[19]^). To determine if alternate promoter usage could explain the expression differences observed in the POLG mutant, log2FC expression changes were examined for each gene expressed by the available promoters, HSP and LSP [Figure 3]. No pattern of promoter-specific effect could be identified.

### Investigation of amino acid properties for mt-tRNAs that showed altered expression by RNA-seq

**Figure 4.**
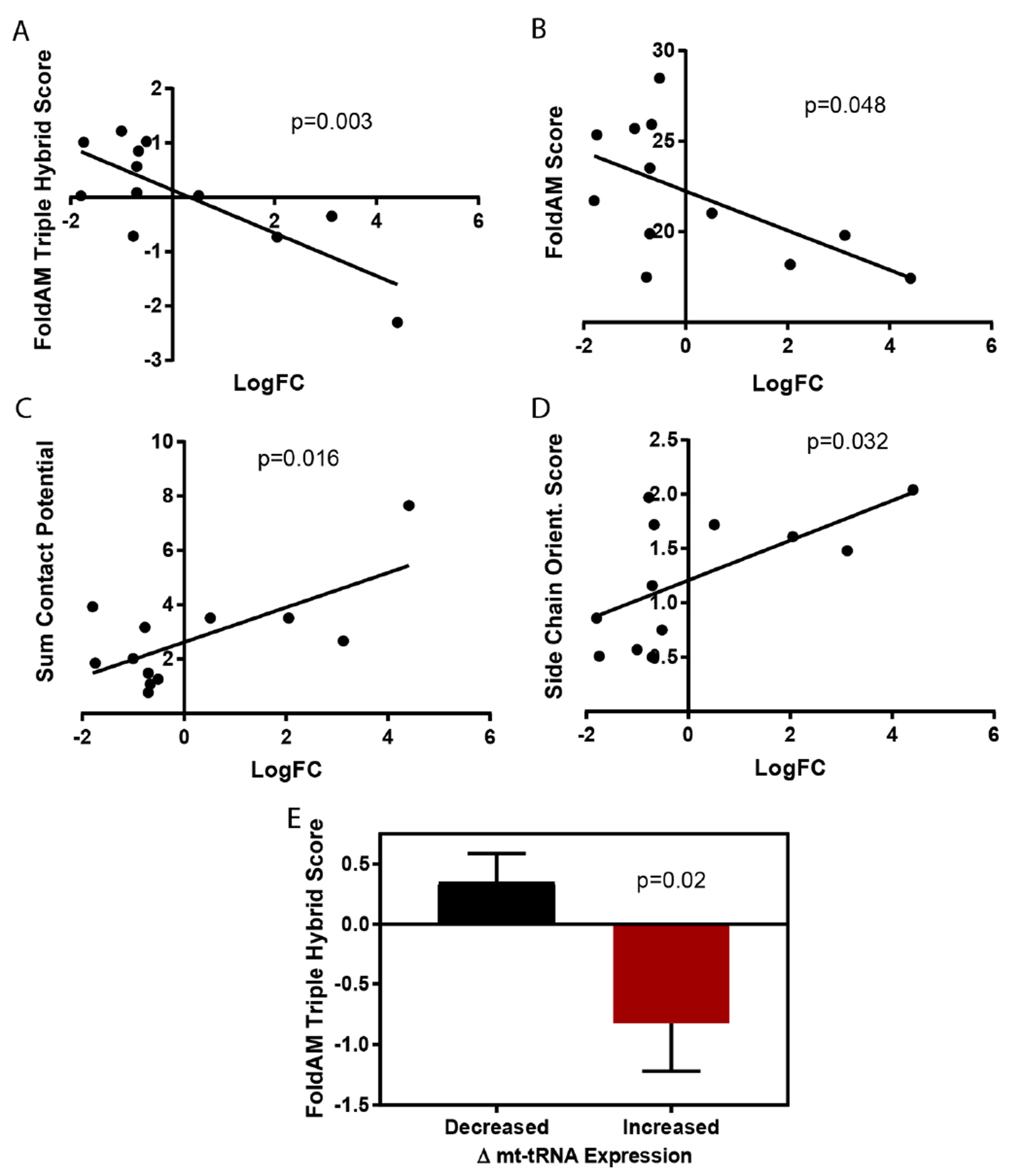
POLG-dependent changes in mt-tRNAs are negatively correlated with amino acid amyloidogenic potential. Log2FC values for significantly altered mt-tRNAs in the POLG mutant (X-axis) were plotted against a series of amino acid characteristic scores (Y-axis) used for predicting amino acid contributions to protein aggregation and beta-amyloid formation (A-D). Linear regression analysis confirms significant negative correlation with FoldAM triple hybrid and regular scoring criteria (A and B). There was a positive correlation with the AA’s sum contact potential value (C) and side chain orientation scores (D). These scores can be indicative of the AA’s propensity to mediate specific protein:protein interactions. As another validation, group analysis (*t*-test) confirmed lower FoldAM triple hybrid score for mt-tRNAs that showed significantly increased *vs*. decreased expression in the POLG mutant (E).

Large bodies of literature report amino acid characteristics relative to the propensity to form protein aggregates and beta-amyloid structures (recent reviews available, e.g.,^[20–22]^). More recently, increased understanding has been gleaned by studying the amino acid residues in protein disordered regions. Amino acid characteristics in these disordered regions appear to strongly influence non-specific protein interactions that give rise to aggregate formation^[23]^. Mt-tRNA fold change values for those significantly altered in the POLG mutant were plotted against a series of amino acid characteristic scores developed for the study of protein aggregation. The fold changes for the mt-tRNAs showed a significant negative correlation with two scores that are indicative of the amino acid’s amyloidogenic potential [Figure 4A and B^[24]^]. As measured by RNA-seq, POLG mutants increased the expression of mt-tRNAs for benign amino acids and decreased the expression of mt-tRNAs for amyloidogenic amino acids. Consistent with this notion, the mt-tRNA fold changes showed a positive correlation to amino acid Contact Potential Sum [Figure 4C^[25]^] and Side Chain Orientation (Figure 4D^[26]^ as reported in^[27]^), which are associated with specific protein interactions. Further, the average FoldAM triple hybrid score was significantly lower for partner amino acids of mt-tRNAs that showed increased expression relative to those that showed decreased expression [Figure 4E].

### Direct measurement of select mt-tRNAs identified as significantly altered by RNA-seq

**Figure 5.**
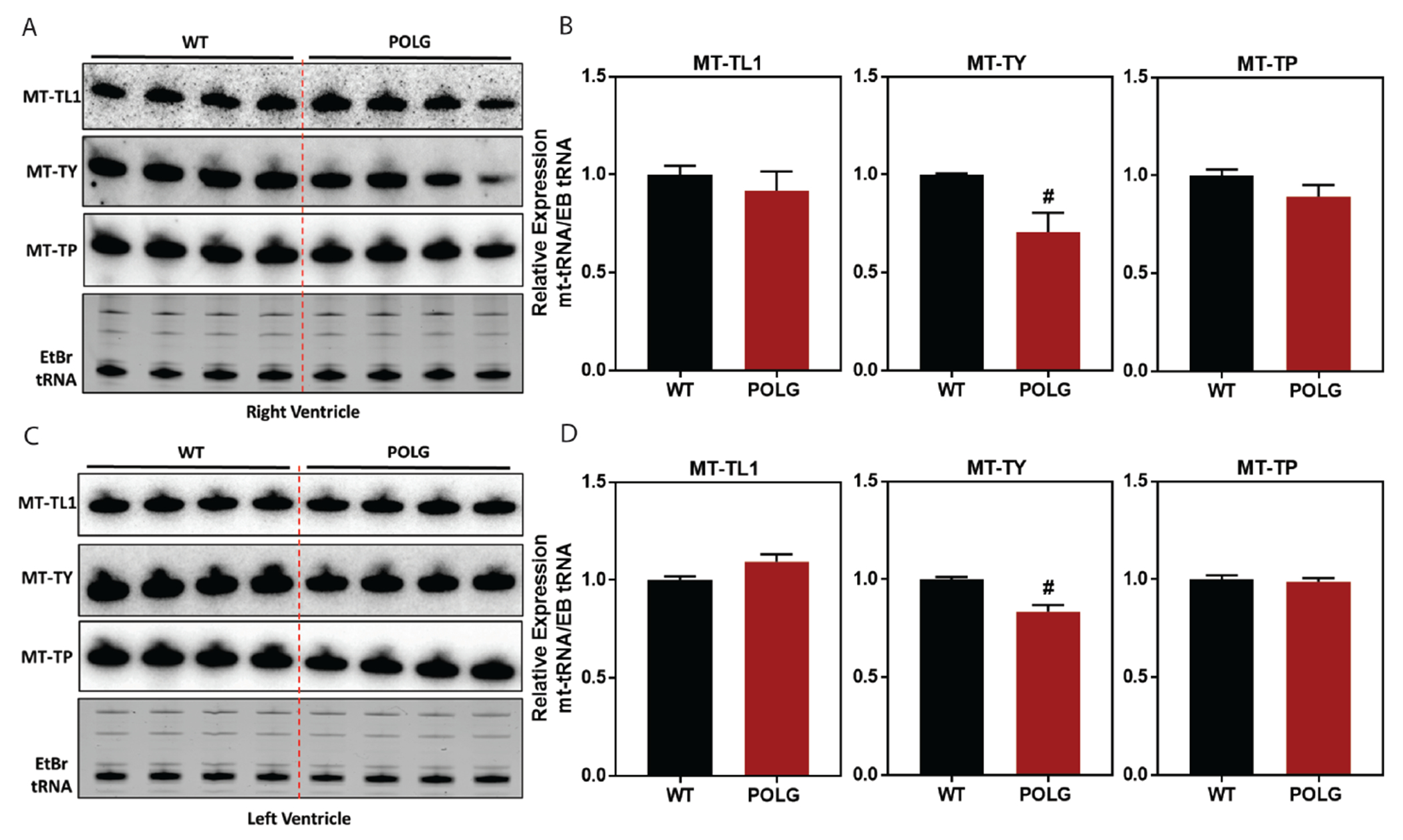
Northern blot analysis of candidate mt-tRNAs identified from RNA-seq analysis. Three mt-tRNAs were selected for measurement by northern blot in cardiac RV and LV tissue: (1)MT-TL1 (control, no change in RNA seq); (2) MT-TY (significantly decreased in RNA-seq); and (3) MT-TP (significantly increased in RNA-seq). In the RV, MT-TY showed significantly reduced abundance in the POLG mutant relative to WT controls (confirming RNA-seq finding) while MT-TP showed no change in abundance between the groups [Northern blots shown in (A) and quantified in (B)]. This pattern of mt-tRNA abundance between WT and POLG mutants was also present in the LV tissue (C and D).

Three mt-tRNAs were selected for measurement by northern blot in cardiac tissue [Figure 5]. Analysis of the left ventricle was included to determine if any patterns observed in the right ventricle [Figure 4A and B] would be conserved in the left ventricle [Figure 4C and D], which has a different developmental origin and experiences different hemodynamics^[28]^. MT-TL1 served as a control as it showed no changes in expression in the POLG mutant by RNA-seq. As expected, MT-TL1 showed no significant expression changes in the POLG mutant. Similarly, MT-TY showed reduced expression in the POLG mutant RV and LV tissues, consistent with RNA-seq. MT-TP, however, did not show any significant change in the POLG mutant, indicating that the dramatically increased MT-TP expression seen in RNA-seq reflected changes in cDNA library generation that were not indicative of actual expression.

## DISCUSSION

There is a clear link between mitochondrial dysfunction and protein aggregation in age-related diseases, including Parkinson’s, Huntington’s, Alzheimer’s, and heart failure^[29–31]^. Nearly all preclinical accelerated aging models show mitochondrial dysfunction and protein aggregation. In the POLG mutant, RNA-seq suggests activation of an adaptive mechanism to limit protein aggregation by shifting mt-tRNA expression towards non-amyloidogenic amino acids. We also suggest that evolution has distributed mt-tRNAs into clusters that allow chromosomal location-dependent regulation, potentially in relation to a mechanism to buffer mitochondrial proteotoxicity. Northern blot recapitulated RNA-seq observed decreased expression of MT-TY, validating the notion that the mitochondria reduce the expression of mt-tRNAs corresponding to amino acids of higher amyloidogenic potential in the POLG mutant. Failure, however, of the northern blot to confirm increased expression of MT-TP complicates interpretation. Given that our RNA-seq measurement is dependent on both polyA capture (mt-tRNAs are poly-adenylated) and cDNA synthesis, we speculate the existence of a post-transcriptional modification on mt-tRNA (either added or removed in the POLG mutant) that dramatically influences the ability of the mt-tRNA to be measured in RNA-seq. Accordingly, increased MT-TP RNA-seq measurement in the POLG mutant may be due to a reduced tRNA modification that improves sequencing efficiency. There are at least 18 types of RNA modifications found at 137 positions in the 22 human mt-tRNAs^[32]^. It is known that m1A9, m1G9, m1G37 and m1A58 modifications confer hard stops in the sequencing of mitochondrial tRNAs^[33]^.

This work does have limitations. First, the current study did not sequence the mitochondrial genome for mutation analysis. Analysis of the mitochondrial mutation landscape in the D257A mutant has been somewhat inconsistent, likely due to both technical challenges and heterogeneity. Using a custom technique termed Mito-seq, Williams et al. conducted an in-depth analysis of mutations in the heart, brain and liver of D257A mutants. While a detailed overlay of mutation data with our expression values was not accomplished, it is important to note that mt-tRNA cluster 1 (MT-TP, MT-TT) closely aligns with an area identified as potentially prone to accumulation of copy number variants or control region multimers in the POLG mutant due to proximity to the origin for replication^[11]^.

Analyzing mitochondrial gene expression by RNA-seq is not common. This may be due to early RNA-seq and microarray analysis pathways being based on nuclear-enriched RNA and the presence of mitochondrial reads indicating poor nuclei isolation. In addition, high mitochondrial read content could indicate apoptosis as cleaved mitochondrial DNA could co-purify with RNA. Another aspect of this discussion is that many commonly used pipelines and practices in transcriptome-wide expression quantification were developed for cancer research where mitochondria content is far lower than in cardiac tissue. For instance, the mitochondrial content of adult cardiac tissue has been reported to range from ~7,000 - ~17,000 mtDNA copies per diploid nuclear genome^[34,35]^. Contaminating mtDNA in the RNA isolation was a consideration in this study, though we do not see it as likely given the DNase treatment of the isolated RNA and non-uniform directionality of altered gene expression in the POLG mutant.

While testing to determine if this phenomenon occurs in human samples remains a future aim, the remarkable conservation of mitochondrial chromosome gene distribution architecture between mice, humans, and even zebrafish suggests that location-dependent regulation of mitochondrial genes would also be conserved. Interestingly, single-celled eukaryotes, such as yeast, show far less mt-tRNA gene distribution across the mitochondrial chromosome, potentially implicating candidate regulatory mechanisms in the evolution of multicellular organisms or tissue/organ specialization.

There has recently been much speculation surrounding the potential ability to stimulate the UPR^mt^ as a therapeutic strategy in multiple disease contexts. Similarly, it is intriguing to ponder: would manipulation of mt-tRNA expression or function based on amyloidogenic/aggregation potential also alter the course of disease? The mechanisms by which mitochondria appear to do this endogenously in the face of severe mitochondrial dysfunction are unknown. Though mitochondrial gene expression was first observed in the 1960s, a detailed understanding of dynamic mitochondrial gene regulation, particularly for mt-tRNAs, is lacking. The basic processes of RNAse-mediated excision from multi-gene/polycistronic transcripts and critical post-transcriptional chemical modifications and amino acid additions are well known (recently reviewed in^[36]^). However, how specific mt-RNAs of different abundance are generated at steady state or in response to stress has not been robustly characterized. It is likely these differences arise through a number of combined mechanisms that could include regulation of transcriptional efficiency (i.e., more expression of genes found early in the transcript), site-specific regulation of RNAse activity for gene excision, regulation of specific mt-tRNA CCA sequence addition, regulation RNA post-transcriptional modifications, and RNA degradation. All of these factors can be impacted by RNA-binding proteins. Conceptually, this regulation could be viewed as mirroring what happens in the nucleus where RNAs can be alternately modified (e.g., splicing and chemical modification) based on locally engaged epigenetic modifiers.

## Supplementary Material

jca-2023-26-SupplementaryMaterials

## Data Availability

Raw and analyzed RNA-seq data for this study are available at the NCBI GEO repository under the accession number GSE199584. Supplementary Table 1 contains a spreadsheet with mitochondria-encoded gene expression values from this study.
